# Aspheric Intraocular Lenses Implantation for Cataract Patients with Extreme Myopia

**DOI:** 10.1155/2014/403432

**Published:** 2014-03-19

**Authors:** Yanwen Fang, Yi Lu, Aizhu Miao, Yi Luo

**Affiliations:** Department of Ophthalmology, Eye & ENT Hospital, Fudan University, 83 Fenyang Road, Shanghai 200031, China

## Abstract

*Objective.* To evaluate the postoperative visual quality of cataract patients with extreme myopia after implantation of aspheric intraocular lenses (IOLs). * Methods.* Thirty-three eyes were enrolled in this prospectivestudy. Eighteen eyes with an axial length longer than 28 mm were included in the extreme myopia group, and the other 15 eyes were included in the nonextreme myopia group. Phacoemulsification and aspheric IOL implantation were performed. Six months after cataract surgery, best-corrected visual acuity (BCVA), contrast sensitivity, and wavefront aberrations were measured, and subjective visual quality was assessed. * Results.* The BCVA improved significantly after surgery for both groups, and patients in the nonextreme myopia group achieved better postoperative BCVA due to better retinal status of the eyes. The evaluation of contrast sensitivity without glare was the same in both groups, whereas patients in the nonextreme myopia group performed better at intermediate spatial frequencies under glare conditions. The two groups did not show a significant difference in high-order aberrations. With regard to subjective visual quality, the composite scores of both groups did not differ significantly. * Conclusions.* Aspheric IOLs provided good visual outcomes in cataract patients with extreme myopia. These patients should undergo careful evaluation to determine the maculopathy severity level before surgery.

## 1. Introduction

The spherical aberration of the cornea is positive and that of the lens is negative in the young, healthy eye, and these differences have a compensatory relationship [1, 2]. Decreased optical quality occurs in the aging eye as the spherical aberration of the lens becomes gradually positive and thus loses its ability to compensate for the corneal aberration, which changes little with increasing age [1–5]. 

Conventional spherical intraocular lenses (IOLs) act as an aging lens in which positive spherical aberration cannot compensate for the corneal aberration. Aspheric IOLs can decrease the total amount of ocular spherical aberration after cataract surgery due to their introduction of negative spherical aberration. It has been shown that aspheric aberration-correcting IOLs effectively reduce ocular aberration and improve contrast sensitivity in patients with age-related cataracts [6–15]. However, little is known about the safety and effectiveness of aspheric IOLs in patients with extreme myopia. Therefore, we performed the current study to evaluate the clinical effects of aspheric IOL implantation in cataract patients with extreme myopia by comparing the objective and subjective visual quality achieved with that achieved in nonextreme myopic eyes.

We chose the MC X11 ASP lens (HumanOptics AG) as our experimental IOL because it is currently the only aspheric IOL with a negative power. The MC X11 ASP lens is a one-piece hydrophilic acrylic IOL with a prolate posterior surface that introduces negative spherical aberration to the ocular system. The overall length of this IOL is 11.0 mm. The optic diameter varies from 5.5 mm to 7.0 mm depending on the power of the IOL. IOLs with a power of 18.0 diopter or less have an optic diameter of 7.0 mm. This IOL can be implanted through a 1.8 mm microincision.

## 2. Methods

This prospective study included 33 eyes of 22 cataract patients (12 males and 10 females) who were scheduled to undergo phacoemulsification and IOL implantation surgery between June 2008 and March 2009 at the Eye & ENT Hospital, Fudan University, China. Inclusion criteria were age-related cataract and age between 45 and 70 years. Exclusion criteria included a history of previous ocular surgery, ocular disorders other than cataract, myopia, or macular degeneration, and patient refusal or inability to maintain followup. Patients were divided into two groups according to their ocular axial length. Those with an axial length longer than 28.0 mm were included in the extreme myopia group, and the others were included in the nonextreme myopia group. Informed consent was obtained from all patients before participation in this study. The study was approved by the Ethics Committee of Eye & ENT Hospital, Fudan University.

All surgical procedures were performed by the same surgeon (Y. L.). After topical anesthesia, a 2.2 mm superior clear corneal incision was made, and a 5.5–6.0 mm continuous curvilinear capsulorhexis was created. After hydrodissection, endocapsular phacoemulsification of the nucleus and cortical aspiration were performed using the Intrepid Micro-Coaxial System on the Infiniti (Alcon Laboratories Inc.). The MC X11 ASP IOL was implanted with the associated special IOL delivery system. The incision was closed by hydration without suture. All surgeries were uneventful and free of intraoperative complications.

Preoperative measurements included visual acuity, intraocular pressure, axial length, and corneal endothelial cell density. At 1 day, 3 days, 2 weeks, and 1 month after surgery, visual acuity and slit-lamp examination were performed. Six months postoperatively, visual acuity, refraction, contrast sensitivity, wavefront aberration, and subjective visual quality were assessed. Complications such as posterior capsule opacification (PCO) and retinal detachment were recorded at the last follow-up visit.

Subjective visual quality was evaluated using the self-administered edition of the National Eye Institute Visual Functioning Questionnaire-25 (NEI VFQ-25) [16]. This 25-item questionnaire was designed to assess vision-related quality of life (VRQL). It includes one general health rating question and 11 vision-targeted subscales as listed in Table 2. Each subscale contains 1 to 4 questions. For each question, the answer was converted to a 0-to-100-point score according to the manual, with higher scores representing better VRQL. Questions within each subscale were averaged to create the subscale score. Patients were also asked whether they experienced problems with glare.

Contrast sensitivity was measured using the contrast glare tester CGT-1000 (Takaci) under mesopic illumination (10 cd/m^2^) at a 350 mm testing distance. Contrast sensitivity was tested with normal pupil and refractive correction. Contrast sensitivity was defined as the reciprocal of the contrast threshold, and this value was converted to log contrast sensitivity for statistical analysis.

After mydriasis, high-order aberrations were measured with the Hartmann-Shack aberrometer (WASCA Analyzer, Carl Zeiss Meditec). Three consecutive measurements were performed on each eye. The lateral coma (*Z*_3_^1^), vertical coma (*Z*_3_^−1^), and spherical aberration (*Z*_4_^0^) as well as the root mean square (RMS) values of the total high-order aberrations and 3rd-order to 7th-order aberrations over a 6.0 mm pupil diameter were automatically calculated by the aberrometer.

Statistical analysis was performed using STATA version 7. The recorded contrast sensitivity values were transformed into log values for analysis. Variables were tested for normality and homogeneity of variances. Independent* t*-test was used to compare demographic data, questionnaire scores, contrast sensitivity, and wavefront aberrations between the two groups. Logarithm of the minimum angle of resolution (log MAR) visual acuity values were tested by the Wilcoxon rank-sum test. The incidence of glare was evaluated with Fisher's exact test. Comparison of Snellen visual acuity before and after surgery was performed using the CMH chi-square test. A *P* value less than 0.05 was considered statistically significant.

## 3. Results

Demographic data for patients enrolled in the study are listed in Table 1. We found no maculopathy in the nonextreme myopia group. However, 7 eyes (38.9%) in the extreme myopia group had preexisting maculopathy before surgery, including 6 eyes with degenerative myopic maculopathy and 1 eye with macular schisis.

**Figure 1:**
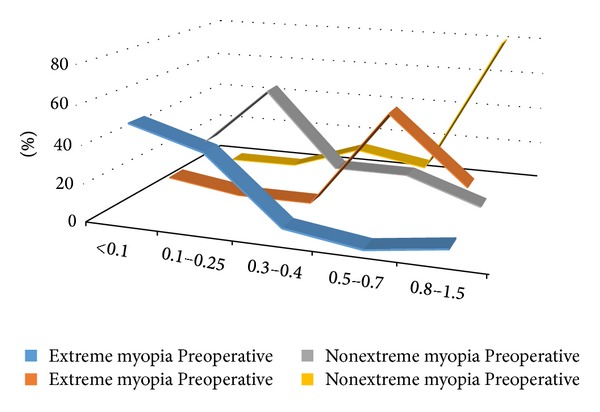
Best-corrected visual acuity before and 3 months after surgery (Snellen visual acuity).

Pre- and postoperative Snellen visual acuities are listed in Figure 1. Six months after surgery, 77.8% of eyes in the extreme myopia group achieved a BCVA of 20/40 or better, including 22.2% of eyes with 20/25 or better. Four eyes demonstrated poor vision of less than 20/60 due to severe macular degeneration or macular schisis. In the nonextreme myopia group, 86.7% of eyes achieved a BCVA of 20/40 or better, including 80.0% of eyes with 20/25 or better. Two eyes had visual acuity of 20/60 and 20/50 due to PCO. The postoperative BCVA of both groups was significantly better than the respective preoperative BCVA values (*χ* ^2^ = 16.36, *P* = 0.0001; *χ* ^2^ = 20.94, *P* = 0.0000), and the nonextreme myopia group performed significantly better than the extreme myopia group (*χ* ^2^ = 5.98, *P* = 0.0144).

Table 2 lists the postoperative NEI VFQ-25 subscale scores of the two groups. The average composite score was not statistically different between the two groups. However, evaluation of the VFQ-25 subscale scores revealed that the nonextreme myopia group performed significantly better than the extreme myopia group in terms of mental health and dependency. In addition, the extreme myopia group showed a trend toward lower social functioning. Data related to driving were not analyzed, because none of the patients in the extreme myopia group and only four patients in nonextreme myopia group drove. Other subscale scores were similar between the two groups.

**Figure 2:**
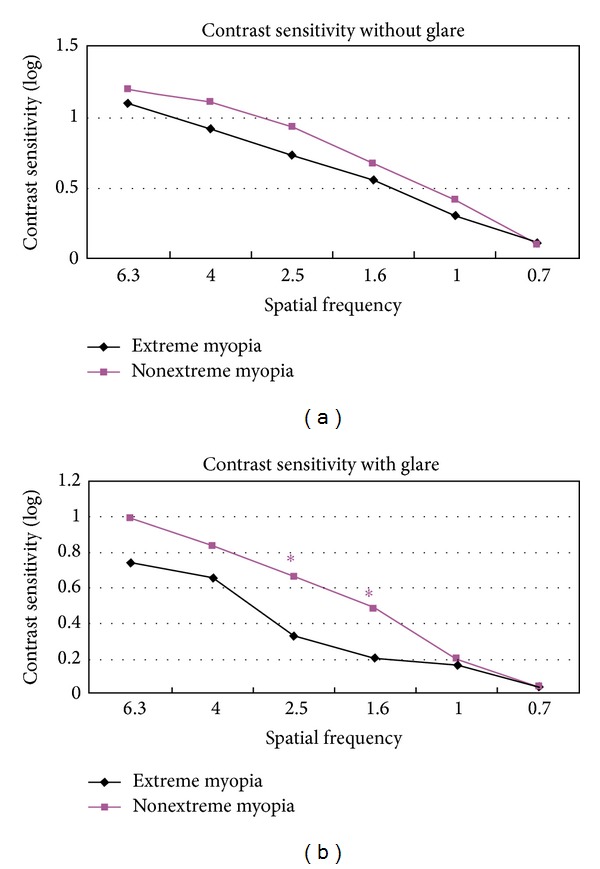
Postoperative contrast sensitivity of the two groups: (a) under nonglare conditions and (b) under glare conditions. *Significant difference between groups.

As illustrated in Figure 2(a), contrast sensitivity without glare did not differ between the groups at each spatial frequency. However, patients in the nonextreme myopia group showed better contrast sensitivity with glare at intermediate frequencies (visual angle of 2.5 degrees, *P* = 0.0277 and 1.6 degrees, *P* = 0.0181) (Figure 2(b)). 

**Figure 3:**
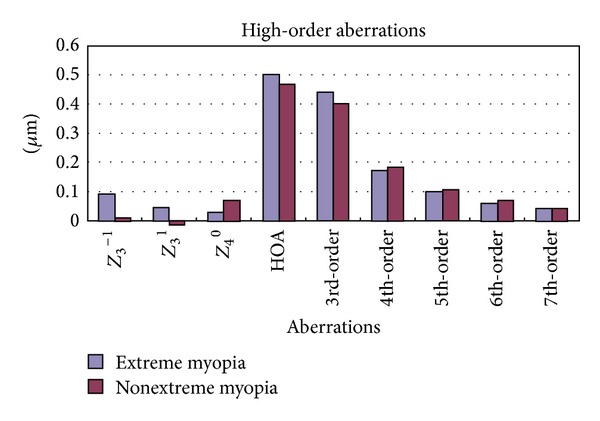
High-order aberrations over a 6 mm pupil diameter. HOA: high-order aberration.

For a 6 mm pupil, the average spherical aberration (*Z*_4_^0^) was 0.03 ± 0.11 *μ*m in the extreme myopia group and 0.07 ± 0.07 *μ*m in the nonextreme myopia group, and total high-order aberrations were 0.50 ± 0.17 *μ*m and 0.46 ± 0.15 *μ*m, respectively. There were no significant differences in lateral coma, vertical coma, spherical aberration, total high-order aberrations, or 3rd-order to 7th-order aberrations (Figure 3).

One patient complained of glare in one eye (5.6%) in the extreme myopia group, and three eyes (20%) were associated with complaints of glare in the nonextreme myopia group, which did not represent a statistically significant difference (*P* = 0.340). The average follow-up period was 6.94 ± 1.47 months (range 6 to 10) in the extreme myopia group and 6.60 ± 1.59 months (range 6 to 12) in the nonextreme myopia group. At the last followup, two eyes in each group (11.1% and 13.3%, resp.) had moderate PCO but did not require further treatment. No retinal detachment occurred in either group. One eye in the extreme myopia group developed capsular block syndrome, and the eye achieved a final visual acuity of 20/30 following anterior capsulotomy.

## 4. Discussion

It is well known that myopia is more common in Asia than in America, Europe, and Africa with a prevalence of 32.3% in the urban adult Chinese population reported by the latest epidemiological study published in 2009 [17]. High myopia prevalence also varies geographically, ranging from 1.7% to 3.3% in Europe, while it affects up to 24% of university students in South-East Asia [18]. In this study, we carried out phacoemulsification and aspheric IOL implantation with low or negative power on cataract patients with extreme myopia. We report the visual performance of aspheric IOLs in terms of functional vision in cataract eyes with extreme myopia in comparison to that achieved in patients with nonextreme myopia. 

Our results show that aspheric IOL implantation provided good visual outcomes in most eyes with extreme myopia, although some patients with extreme myopia had relatively worse retinal status than nonmyopia patients. When the seven eyes with maculopathy in the extreme myopia group were excluded from the data analysis, postoperative visual outcomes were similar between the two groups (*χ* ^2^ = 1.45, *P* = 0.2288). For the seven excluded eyes, visual acuity improved after surgery anyway: one improved from hand moving to 20/400, one improved from 20/2000 to 20/250, and the other 5 improved 2 to 8 lines (Snellen chart). In these patients, postoperative vision was mostly determined by the severity of the maculopathy.

After objective visual acuity measurements, we assessed the subjective visual quality of the patients using the NEI VFQ-25. This questionnaire was designed to capture the impact of visual problems on physical functioning, emotional well-being, and social functioning [16]. It has been widely used to evaluate health-related quality of life in patients with various eye diseases and treatments [19–25]. The score from the extreme myopia group was similar to that reported by Lin et al. [23], who implanted the aspheric IQ IOL (SN60WF, Alcon Laboratories Inc., Fort Worth, TX, USA). Interestingly, although the nonextreme myopia group had better postoperative BCVA in the present study, the two groups reported similar subscale scores for near and distance activities; that is, the patients' perception of their visual function was similar. This might be attributed to the preoperative visual status of the patients with extreme myopia who always had poor visual function. Therefore, the removal of the cataract along with the correction of the refractive errors likely resulted in a high level of patient satisfaction in terms of visual outcome. However, the subscale scores of mental health and dependency were significantly lower in this group, suggesting that these patients worried more about their eyesight and that their quality of life was more affected by vision.

Contrast sensitivity is a robust indicator of functional vision [26]. In this study, we found that patients with nonextreme myopia had better contrast sensitivity at intermediate spatial frequencies under glare conditions. These data are in agreement with Stoimenova's findings [27], which showed that myopes exhibited reduced sensitivity to contrast in comparison to emmetropes and that contrast sensitivity decreased with an increasing degree of myopia. This difference may contribute to the aberrations of the myopic eyes or functional/morphologic changes in the retina of myopic eyes.

We also compared high-order aberrations between the groups and found that postoperative ocular spherical aberration (*Z*_4_^0^) was near zero in the two groups, which fulfilled the aim of compensating the corneal spherical aberration with an IOL. High-order aberrations of the two groups did not show a significant difference. The total effect of all monochromatic optical aberrations represents the optical quality of the eye [26]. Because the spherical and cylindrical refractive errors were fully corrected and high-order aberrations were similar between groups, the optical quality of the ocular system in the extreme myopia group was as good as that in the nonextreme myopia group. However, the extreme myopia group showed worse visual acuity and contrast sensitivity, mainly because of the poor retinal status. In the absence of macular degeneration, the eyes with extreme myopia would have achieved visual acuity as good as that of eyes with nonextreme myopia. Therefore, evaluation of the retinal status before surgery is very important.

The complications experienced by patients in the two groups were comparable overall. Glare disturbance occurred in 5.6% of patients in the extreme-myopia group and 20% of patients in the nonextreme myopia group, and this difference was consistent with the results of previous studies. Franchini [28] reported that glare occurred in 20% of patients who received the aspheric Tecnis Z9000 IOL (Abbott Laboratories). In the study by Johansson et al. [29], 21.1% of patients who received an Akreos Adapt AO IOL (Bausch & Lomb Laboratories Inc.) in one eye and a Tecnis Z9000 IOL in the other eye experienced glare. In the current study, the extreme myopia group experienced relatively less glare. A possible cause may be that the macular disorder of some of the eyes was so severe that the patients could not detect the glare. In terms of the PCO and retinal detachment, no significant differences were found between the groups.

The aspheric IOL used in this study was the MC X11 ASP, which features a large optic diameter (7.0 mm) for middle to low IOL powers. It has been shown that greater optic diameter can reduce postoperative glare [30, 31]. Larger optic diameter can also facilitate postoperative peripheral retinal examination and treatment, because eyes with high myopia are at high risk of lattice degenerations, retinal holes and tears, and retinal detachment [18]. Because of the larger optic diameter, a larger capsulorhexis of 6.0 to 6.5 mm was required. One patient developed capsular block syndrome, which may have been caused by a relatively small capsulorhexis opening.

In conclusion, aspheric IOLs can provide good visual outcomes for cataract patients with extreme myopia. Visual results were comparable to those observed in patients with nonextreme myopia. Also, preoperative evaluation of the severity of maculopathy is very important and should be carefully performed in eyes of patients with extreme myopia before surgery.

## Figures and Tables

**Table 1:** Demographic data of patients enrolled in this study.

Characteristic	Extreme myopia	Nonextreme myopia	*P* value
Number of patients/number of eyes	11/18	11/15	/
Age (years)	53.64 ± 6.31	59.36 ± 9.96	0.1230
Axial length (mm)	31.64 ± 1.62	24.97 ± 1.31	0.0000
Predicted IOL power (diopter)	−0.92 ± 4.71	16.13 ± 4.47	0.0000
Implanted IOL power (diopter)	3.22 ± 4.14	17.27 ± 3.49	0.0000
Preoperative BCVA (log⁡MAR)	1.15 ± 0.60	0.86 ± 0.45	0.0693
Postoperative BCVA (log⁡MAR)	0.35 ± 0.36	0.06 ± 0.18	0.0011
Postoperative spherical equivalent	−2.34 ± 1.20	−1.39 ± 1.00	0.0211

*Note*. IOL: intraocular lens; BCVA: best-corrected visual acuity.

**Table 2:** Postoperative NEI VFQ-25 subscale scores.

Subscales	Extreme myopia	Nonextreme myopia	*P* value
General health	45.45 ± 24.54	47.73 ± 17.52	0.8051
General vision	67.27 ± 18.49	69.09 ± 10.44	0.7793
Ocular pain	78.41 ± 13.80	72.73 ± 17.52	0.4080
Near activity	70.83 ± 20.16	76.52 ± 17.80	0.4915
Distance activity	72.35 ± 28.40	83.33 ± 17.08	0.2847
Social functioning	81.82 ± 14.10	92.05 ± 8.43	0.0522
Mental health	43.75 ± 22.36	65.34 ± 18.83	0.0236*
Role difficulties	53.41 ± 29.10	55.68 ± 25.23	0.8468
Dependency	54.55 ± 25.92	85.61 ± 11.24	0.0016*
Driving	/	/	/
Color vision	90.91 ± 16.86	100.00 ± 0.00	0.0888
Peripheral vision	85.00 ± 12.91	86.36 ± 17.19	0.8407

Composite score	69.28 ± 14.20	78.47 ± 9.30	0.0879

*Significant difference between groups.
